# The Role of Small Woodland Remnants on Ground Dwelling Insect Conservation in Chaco Serrano, Central Argentina

**DOI:** 10.1673/031.013.4001

**Published:** 2013-05-13

**Authors:** María Laura Moreno, María Guadalupe Fernández, Silvia Itati Molina, Graciela Valladares

**Affiliations:** 1 Centro de Investigaciones Entomológicas de Córdoba, F.C.E.F. y N., Universidad Nacional de Córdoba, Av. Vélez Sarsfield 266 (X50I6GCA), Córdoba, Argentina; 2 Instituto Multidisciplinario de Biología Vegetal (CONICET-Universidad Nacional de Córdoba), Av. Vélez Sarsfield 1611 -(X5016GCA), Córdoba, Argentina

**Keywords:** epigaeic insects, feeding guilds, habitat fragmentation, subtropical forest

## Abstract

Many terrestrial ecosystems are changing due to extensive land use and habitat fragmentation, posing a major threat to biodiversity. In this study, the effects of patch size, isolation, and edge/interior localization on the ground dwelling insect communities in the Chaco Serrano woodland remnants in central Argentina were examined. Sampling was carried out in December 2003 and March 2004 in nine remnants (0.57 to 1000 hectares) using pitfall traps. In total, 7071 individuals representing 12 orders and 79 families were recorded. The taxonomic composition of these communities was linked to remnant size. Insect abundance increased (as did their richness, albeit marginally) as remnant area decreased, with no significant effects of isolation or edge/interior localization on abundance, richness, or diversity. No differential area effects were observed when abundance and richness of predators, scavengers, and herbivores were compared. Thus, ground insect communities in fragmented Chaco Serrano seem to respond mainly to patch level, rather than to within-patch (edge effects) or landscape (isolation) level variables. These results suggest that small Chaco Serrano remnants, by supporting larger ground-dwelling insect assemblages, may play an important role from a conservation viewpoint.

## Introduction

Habitat fragmentation, i.e., the transformation of an originally large habitat into smaller and isolated remnants embedded in a matrix with different land use, has been recognized as a major threat to biodiversity (Fahrig 2003). Remnant area and isolation are expected to affect population abundance and diversity (Ewers and Didham 2006). According to the island biogeography theory (MacArthur and Wilson 1967), smaller and more isolated remnants should sustain fewer species than larger and less isolated sites, as a consequence of lower immigration and higher local extinction rates (Fujita et al. 2008). Also, metapopulation theory (Hanski and Gilpin 1991) predicts that populations in habitat patches periodically go extinct and are recolonized by individuals migrating from other remnants across the matrix (Davies et al. 2001), therefore increasing isolation should result in reduced probability of colonization. Another important feature of fragmented habitats is the difference in abiotic conditions, including temperature, humidity, light, and wind speed, at the edge of remnants in comparison with their interior (Saunders et al. 1991; Murcia 1995; Laurance et al. 2007). These conditions could strongly affect small and ectothermic animals such as insects (Grimbacher et al. 2006). Moreover, conditions at the edge habitat could selectively impair survival of some species while facilitating invasion by opportunist/generalist species from the matrix (Laurance et al. 2002; Tscharntke et al. 2002a; Ewers and Didham 2006), thus resulting in higher diversity and altered composition.

Insects represent the major group in terms of terrestrial biodiversity, accounting for over 75% of all known animal species (Speight et al. 2008). Because of their diversity, abundance, easy sampling, and rapid response to environmental changes, insects are useful bio-indicators of habitat degradation (Bolger et al. 2000; Cagnolo et al. 2002) and have therefore been widely used to study the effects of habitat fragmentation (Debinsky and Holt 2000; Hunter 2002; Grimbacher et al. 2008). In particular, the study of epigaeic or ground dwelling insects has important functional implications because of the insects varied ecological roles, as they include predators, scavengers, and herbivores (Didham 1996).

Heterogeneous responses to remnant size have been reported for ground insect species in fragmented woodlands, ranging from positive (Didham et al. 1998; Fujita et al. 2008) to negative relationships (Davies et al. 2001; Yaacobi et al. 2007) between abundance, richness or diversity, and remnant area. Also, contrasting responses have been found for edge (Grimbacher et al. 2006; Sobrinho and Schoereder 2006) and isolation effects (Yaacobi et al. 2007). However, little is known about habitat fragmentation effects at higher taxonomic levels such as family (Grimbacher et al. 2008), which have proved useful to evaluate insect responses in disturbed habitats (Basset et al. 2004). Family taxonomic level is fairly often used in analyses of perturbation effects on ground dwelling insects, with limitations for inferences regarding within-family variability (correlation with environmental conditions may be masked by the differential responses of different species from the same family) being compensated by the possibility of assessing broader community trends (e.g., Zilihona and Nummelin 2001; Yu et al. 2006; Bennet and Gratton 2012).

The functional diversity of epigaeic insects allows for the consideration of feeding guilds as a complementary analysis to that of taxonomic diversity. The analysis of feeding guilds focuses on “what organisms do” by grouping together species that exploit the same resources in the same way (Blaum et al. 2011). This approach is critical for understanding the potential consequences of biodiversity for ecosystem processes, and helps generalization of the results in comparison with taxonomically centered studies (Blaum et al. 2011). Not all feeding guilds are expected to be affected by habitat fragmentation in the same way. The trophic rank hypothesis (Zabel and Tscharntke 1998; Holt et al. 1999) predicts that species at higher trophic levels, like predators, are more vulnerable to extinction following a perturbation, such as habitat fragmentation. In turn, changes at higher trophic levels could trigger further community changes that might affect the system dynamics. However, few studies have considered the effects of subtropical forest fragmentation on ground dwelling insects in terms of feeding guilds (e.g., Lange et al. 2011), since research has usually been focused on restricted groups of species, mostly within Coleoptera (Davies et al. 2001; Grimbacher et al. 2008).

In central Argentina, human activities have led to a 94% reduction of the Chaco Serrano woodland, a district within the extensive South American Chaco forest, resulting in isolated patches within a predominantly agricultural matrix (Zak and Cabido 2004). In this system, habitat fragmentation has been shown to affect plant diversity and reproduction (Aguilar and Galetto 2004; Cagnolo et al. 2006; Galetto et al. 2007), herbivory and parasitism rates (Valladares et al. 2006), species richness of insect herbivores and parasitoids (Cagnolo et al. 2009), and even food web structure (Valladares et al. 2011). In our study, the communities of ground dwelling insects associated to remnants of Chaco Serrano are examined in a fragmented landscape.

Whether abundance, diversity, and taxonomic composition of ground dwelling insect families are related to remnant area, isolation, or within-remnant location (edge/interior) is examined. Furthermore, a functional approach is included, by partitioning the studied communities in feeding guilds, in order to consider the possibility of differential risk from habitat fragmentation in relation to particular ecological processes. In particular, abundance, richness, and diversity of ground dwelling insect families were expected to be positively related with remnant area and negatively with isolation. Also, there were expected to be more abundant and diverse insect assemblages at the edge of remnants in comparison with their interior. Finally, predators were expected to be most sensitive to habitat fragmentation.

## Methods

### Study area

This study was conducted within the Chaco Serrano District, in Central Argentina (Luti et al. 1979). The average annual rainfall in the region is about 750 mm, concentrated in the warm season (October–April), with maximum and minimum temperatures of 26° C and 10° C, respectively (Luti et al. 1979; Moglia and Jiménez 1998). The characteristic vegetation is a low, open woodland, with a tree layer (8– 15 m high) dominated by *Aspidosperma quebracho-blanco* Schltdl., *Prosopis* spp., *Zanthoxylum coco* Gillies ex Hook. f. and Arn., and *Lithrea molleoides* (Vell.) Engl.; shrubs (1.5–3 m), such as *Celtis pallida* Torr. and *Acacia* spp.; herbs and grasses (0–1 m), and many vines and epiphytic bromeliads (Cabido et al. 1991). At present, this vegetation is reduced to isolated remnants (Zak et al. 2004) embedded in a predominantly agricultural matrix.

**Figure 1.**
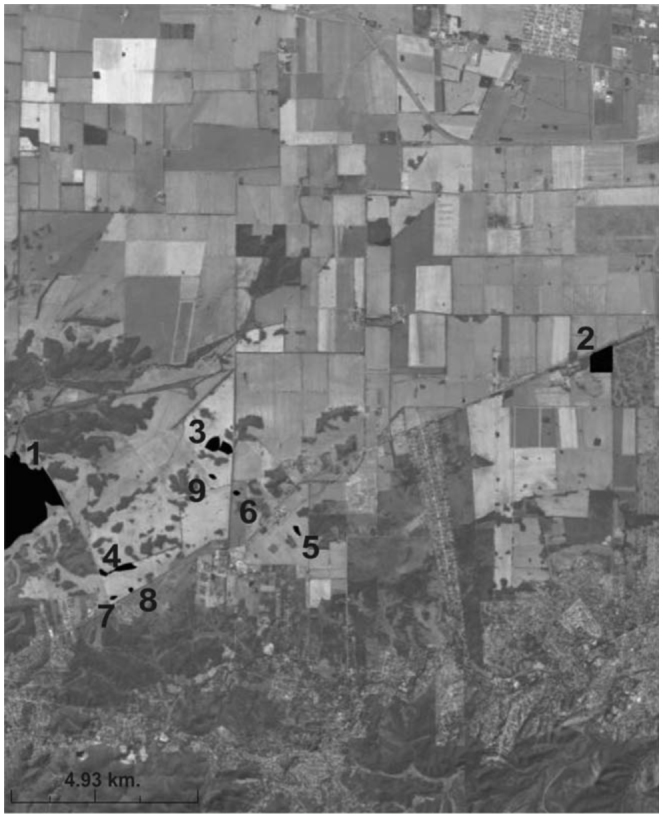
Location of study remnants (in black) in Chaco Serrano forest, Central Argentina. High quality figures are available online.

Nine remnants, ranging in size from 0.57 hectares to over 1000 hectares, were selected (Figure 1) within an area located between 31° 10′ and 31° 30′ S and 64° 00′ and 64° 30′ W, with an elevation of 500 to 600 m. The degree of isolation was estimated by the nearest neighbor method (Krebs 1999). All remnants had been isolated for at least 30 years in a matrix dominated by wheat in winter and soybean or maize in summer.

### Sampling procedures

The insects were captured using pit-fall traps (10 cm diameter, 15 cm deep) containing a 20% solution of ethylene glycol. The traps were exposed 5 to 7 days at each of two sampling dates, December 2003 and March 2004. At each remnant, three traps were placed 5 m apart from each other and within 5 m of the tree line indicating the boundary of the forest (edge location), and three more traps were placed in a row parallel to the former but at least 20 m from the forest border (interior location). All insects collected were counted and identified to family level. The following variables were calculated: abundance (number of individuals), richness (number of insect families), and diversity (Shannon-Weaver index). This index was calculated as: H' = -Σp*_i_*.lnp*_i_*, where p_i_ is the proportion of individuals found in the *i*th families (Magurran 2004). Because Shannon entropies index gives the uncertainty rather than diversity, Shannon values were converted to effective number of species (e^(H')^) (Jost 2006). The following feeding guilds were considered for functional analysis: scavengers, herbivores, and predators. When more than one guild was represented within a family, the predominant habit was considered (Borror et al. 1997).

### Data analysis

The degree of similarity in the taxonomic composition of ground insect communities associated to the various remnants was explored by performing a correspondence analysis and subsequent correlation analyses on insect family abundance data. Spearman correlations were employed between community position for the first two correspondence analysis axes, and remnant area or isolation, in order to assess the importance of habitat fragmentation variables on community taxonomic arrangement. A Mantel Test was performed to provide further insight into spatial effects on insect community composition by considering whether communities most closely resemble those in nearer remnants. The Mantel Test examines the null hypothesis of non-concordance between two distance matrices (Legendre and Legendre 1998). The Bray-Curtis distance matrix based on insect family abundance data was compared to a matrix based on geographical distance (km) between sampling sites, using the Mantel's asymptotic approximation for statistical significance.

To analyze possible effects of habitat fragmentation on the insect communities, a linear mixed model was performed (Pinheiro and Bates 2000), with abundance (N_T_), richness (S), or diversity of insect families as response variables, location (edge/interior), remnant area, and isolation as fixed effects, and site (remnant) as random effect. Interactions between fixed effects were evaluated. Non-significant interactions were removed in order to obtain the most parsimonious model. The statistical analyses were performed using the software R 2.11.0 (R Development Core Team 2010).

The effects of remnant area on the different feeding guilds were compared by means of analysis of covariance (ANCOVA) considering abundance and richness of each feeding guild as response variables, feeding guild as the fixed effect, and remnant area as the covariable. A significant interaction with remnant area would indicate different slopes and thus differential area effects for the groups considered (Zar 1996).

**Table 1.**
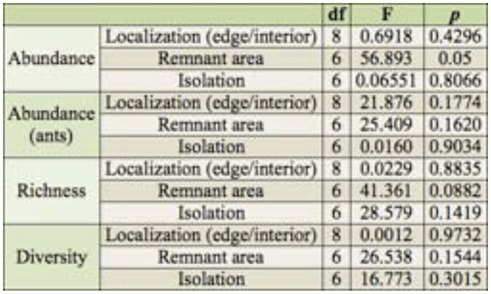
Results of linear mixed models examining the relationship between edge/interior localization, remnant area and isolation vs. abundance, richness, and diversity of ground-dwelling family insects in nine Chaco Serrano remnants.

Statistical analyses were based on the average number of individuals per trap per capture day. After checking data distribution, values were log x+1 (insect abundance) or log transformed (family richness and remnant size) in order to achieve normal distribution. Ants (Hymenoptera: Formicidae) were analyzed separately because of their extremely high abundance coupled with social and gregarious behavior.

## Results

### Taxonomic composition

In total, 7071 insects representing 12 orders and 79 families were collected (see Appendix).

**Figure 2.**
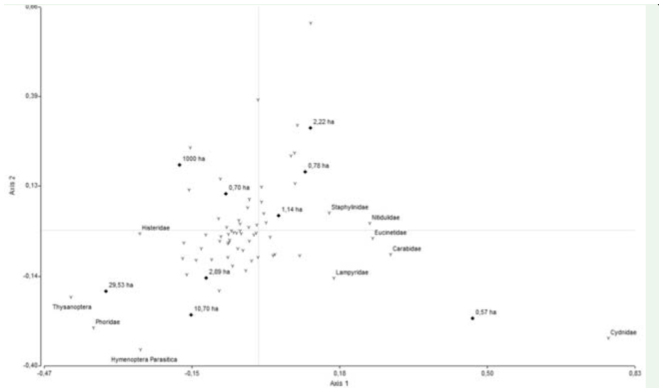
Correspondence analysis of ground dwelling insect communities from nine remnants of Chaco Serrano. Squares represent forest remnants, triangles represent insect families. Figures next to each square indicate remnant size (in hectares). Only families with the strongest association with the first axis are named. High quality figures are available online.

Slightly over half of the variation in taxonomic composition of the studied insect communities was explained in the correspondence analysis, with the first axis accounting for 29.78% and the second axis 24.92% of that variation (Figure 2). Only the first correspondence analysis axis was strongly negatively correlated with remnant area (r = -0.83, p = 0.02), whereas no axes were correlated with isolation. Staphilinidae, Nitidulidae, Eucinetidae, Carabidae, Cydnidae, and Lampyridae were the families with stronger positive association to the first axis, i.e., to smaller remnants, whereas Hymenoptera parasitica, Thysanoptera, Phoridae, and Histeridae were closely associated to larger remnants

Moreover, the Mantel Test showed no significant compositional turnover with geographic distance (r = -0.31, *p* = 0.29).

### Abundance, richness, and diversity of insect families

**Figure 3.**
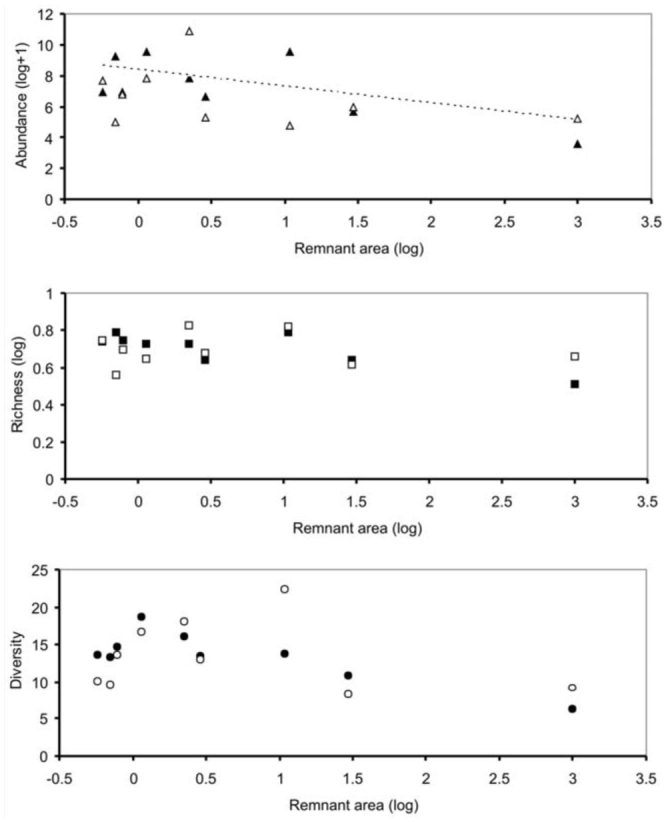
Relationship between abundance (a), richness (b), and diversity (c) of ground dwelling insects vs. remnant area in Chaco Serrano. Regression lines based on linear mixed models (see Table 1). Open symbols = interior, filled symbols = edge. High quality figures are available online.

Abundance and richness of insect families decreased as remnant area increased, although results for the latter variable were only marginally significant (Table 1, Figure 3). Community diversity (Table 1) and ant abundance (Table 1) were independent of remnant area. Moreover, there were no effects of isolation or edge/interior location on insect abundance, richness, or diversity (Table 1).

### Functional composition

**Figure 4.**
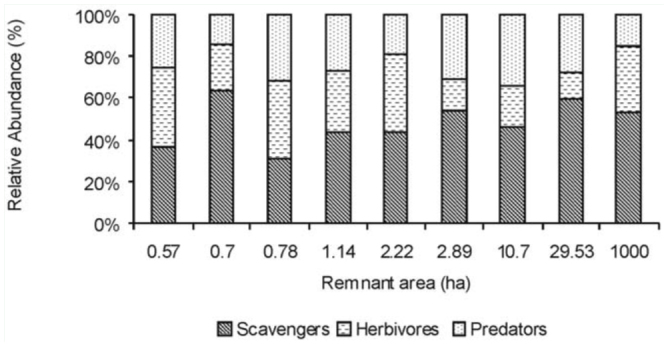
Relative representation of ground dwelling insect feeding guilds in nine remnants of Chaco Serrano. High quality figures are available online.

Regarding functional composition (Figure 4) of the ground dwelling communities, scavengers were the most abundant (49 ± 2% of collected insects) and diverse (36% of families) feeding guild, followed by herbivores (28 ± 2.38% individuals, 32% families) and predators (22 ± 1.14% individuals, 20% families).

**Table 2.**
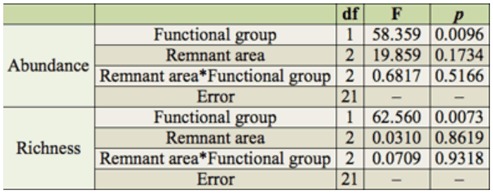
Results from analysis of covariance testing for differential relationships in abundance and richness of three feeding guilds (predators, herbivores, scavengers) vs. remnant area in Chaco Serrano.

Feeding guild significantly influenced insect abundance and richness, but did not show differential relationships with area (Table 2).

## Discussion

According to the results of our study, differences in abundance and taxonomic composition of ground dwelling insect communities were linked to forest area, with no obvious edge or isolation effects.

Which insect families were present in a particular forest remnant seemed to be at least partly determined by the size of the remnant. Different factors could be operating at different spatial scales, as observed for other ground dwelling insect communities (e.g., Barton et al. 2009) and could be related to environmental heterogeneity or to insect traits such as dispersal ability, body size, and food requirements.

Contrary to expectations from island biogeography theory and metapopulation theory, ground dwelling insects in this fragmented Chaco Serrano landscape were more abundant and, albeit marginally, richer in smaller remnants. Negative relationships between abundance and habitat area have also been reported in other studies on epigaeic insects (e.g., Davies et al. 1998; Grez et al. 2004; Henríquez et al. 2009). Such trends could reflect colonization by habitat generalists arriving from or through the matrix, as small remnants would be the most likely place for habitat generalists to invade (Halme and Niembelä 1993). In other cases, similar patterns of abundance/richness have been shown to be related to resource availability in systems where small remnants showed higher habitat heterogeneity or plant diversity (Tscharntke et al. 2002b; Jonsson et al. 2009). However, previous studies have found lower plant richness in smaller Chaco Serrano remnants (Cagnolo et al. 2006). Since capture in pitfall traps depends on insect mobility (Perner and Schueler 2004), higher abundance could also result from easier movements in a less restrictive environment, e.g., if ground level vegetation structures were simpler in smaller remnants.

Communities at remnant edges did not differ from those found deeper in the forest, either in terms of abundance, richness, or diversity of epigaeic insect families. It is possible that inputs from the matrix may have numerically compensated for the absence of some interior specific insects at the edge. For example, in a forest-clearcut ecotone in China, abundance, richness, and diversity of ground dwelling beetle families were similar in forest interior and edge, despite the edge hosting insects from both the forest and the clearcut matrix (Yu et al. 2006). Obviously, patterns observed at family taxonomic level may mask species-specific responses to environmental conditions. In this sense, various studies have shown species-specific and trait-related responses to the interior/edge situation (Didham et al. 1998; Davies et al. 2001; Lövei et al. 2006), which might be overlooked when considering general community trends. Moreover, edge-related microclimatic changes in some systems can penetrate more than 30 m into the interior (Laurance et al. 2002), thus the distance between edge and interior in samples from our study (20 m) might be deemed insufficient to detect edge effects. Nonetheless, in a previous study, the same distance was enough to show edge-related differences in herbivory and parasitism rates in Chaco Serrano remnants (Valladares et al. 2006).

The consequences of habitat fragmentation effects on particular functional groups or feeding guilds could be important because of their possible impact on ecosystem functioning. However, in coincidence with other studies on ground dwelling insects (Yu et al. 2006; Grimbacher et al. 2008), no differential relationship was found in our study between feeding guilds and habitat fragmentation. Contrary to expectations, predators did not appear to be more sensitive to habitat loss than herbivore or scavengers insects, therefore the results did not support the trophic position hypothesis (Kruess and Tscharntke 2000; Holt 2002). Despite the variations in abundance and species number reported above, communities of ground dwelling insects along a gradient of remnant sizes appear thus to be similar in terms of the ecosystem functions they perform.

In summary, by applying a multilevel approach (Thornton et al. 2011) simultaneously considering within-patch (edge effects), focal patch (area), and landscape level (isolation) variables, it was found that ground insect communities in fragmented Chaco Serrano respond mainly to the patch level by increasing their abundance (and, in a lesser degree, their richness) in smaller remnants. The lack of isolation or edge effects in this study corroborates the importance of area as the main factor affecting biodiversity in fragmented systems (Fahrig 2003). These results suggest small Chaco Serrano remnants may play an important role from a conservation viewpoint by supporting larger ground dwelling insect assemblages, and preserving remnants of various sizes would be favorable for insect conservation in this subtropical forest.

## Figures and Tables

**Appendix.**
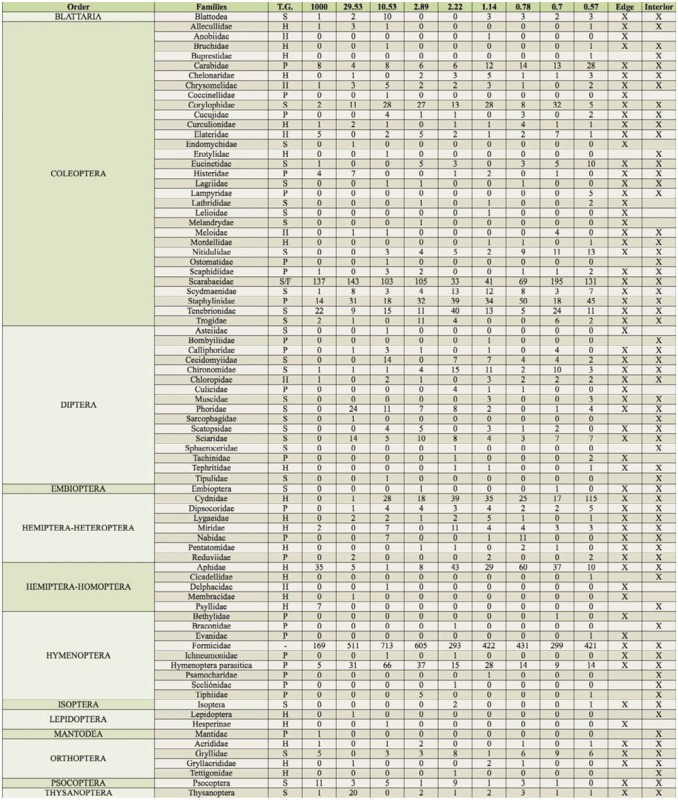
Total abundance of insects families captured in nine remnants of Chaco Serrano between December 2003 and March 2004 in Chaco Serrano woodland. T.G. indicates taxonomic group (S = scavengers, H = herbivores, P = predators), and X indicates presence of insect in edge/interior localization.
